# Low risk of complications in patients with first-time acute uncomplicated diverticulitis

**DOI:** 10.1007/s00384-017-2912-7

**Published:** 2017-10-16

**Authors:** Abbas Chabok, Kalle Andreasson, Maziar Nikberg

**Affiliations:** Departments of Surgery and Centre for Clinical Research, Uppsala University, Västmanland’s Hospital, Västerås, Sweden

**Keywords:** Diverticulitis, Acute uncomplicated diverticulitis, Recurrence, Outpatient management, Antibiotics

## Abstract

**Purpose:**

First-time acute uncomplicated diverticulitis (AUD) has been considered to have an increased risk of complication, but the level of evidence is low. The aim of the present study was to evaluate the risk of complications in patients with first-time AUD and in patients with a history of diverticulitis.

**Methods:**

This paper is a population-based retrospective study at Västmanland’s Hospital, Västerås, Sweden, where all patients were identified with a diagnosis of colonic diverticular disease ICD-10 K57.0–9 from January 2010 to December 2014. The records of all patients were surveyed and patients with a computed tomography (CT)-verified AUD were included. Complications defined as CT-verified abscess, perforation, colonic obstruction, fistula, or sepsis within 1 month from the diagnosis of AUD were registered.

**Results:**

Of 809 patients with AUD, 642 (79%) had first-time AUD and 167 (21%) had a previous history of AUD with no differences in demographic or clinical characteristics. In total, 16 (2%) patients developed a complication within 1 month irrespective of whether they had a previous history of diverticulitis (*P* = 0.345). In the binary logistic regression analysis, first-time diverticulitis was not associated with increased risk of complications (OR 1.58; CI 0.52–4.81). The rate of antibiotic therapy was about 7–10% during the time period and outpatient management increased from 7% in 2010 to 61% in 2014.

**Conclusions:**

The risk for development of complications is low in AUD with no difference between patients with first-time or recurrent diverticulitis. This result strengthens existing evidence on the benign disease course of AUD.

## Introduction

The traditional treatment for acute uncomplicated diverticulitis (AUD) was challenged for the first time by a Swedish randomized trial, the AVOD study (Swedish acronym for “antibiotics in uncomplicated diverticulitis”) [1]. A low risk of complications in computed tomography (CT)-verified AUD was found regardless of antibiotic therapy. The results of the AVOD study have been confirmed by several prospective and retrospective studies from Nordic countries [2–4]. A majority of patients with AUD may be treated as outpatients without antibiotics [5]. Even though the risk of complications after an episode of AUD is low, some patients will develop a complication (2–5%) [2–4]. In some studies, patients with first-time AUD are considered to have an increased risk of complications compared with AUD patients with a history of diverticulitis, but the evidence level is very low [6, 7]. The AVOD study included all patients with AUD and was replicated by another randomized trial with the same results; however, only patients with first-time AUD were included [8]. Strict patient selection in randomized studies is a drawback and further studies in a population-based setting are necessary for external validity.

The aim of the present study was to evaluate the risk of complications in a large population-based cohort of patients with first-time AUD and in AUD patients with a history of diverticulitis.

## Methods

This population-based retrospective study was undertaken at the Department of Surgery at the Västmanland’s Hospital, Västerås, Sweden, which serves a population of 260,000 inhabitants. The patients were identified according to the International Classification of Diseases (ICD)-10 in the hospital records system in connection with a discharge either from the surgical department or from the emergency department for those managed as outpatients. All patients with the diagnosis of colonic diverticular disease ICD-10 K57.0–9 from January 1, 2010 to December 31, 2014 were identified. The records of all patients were surveyed and patients with a CT-verified AUD were included in the study. The definition of AUD was diverticulitis without complications such as abscesses, perforation, colonic obstruction, or fistula verified by CT scan imaging.

A complication was defined as CT-verified abscess, perforation, colonic obstruction, or fistula within 1 month from the diagnosis of AUD. A recurrence was defined as a diverticulitis 1 month after discharge.

A predefined protocol was used: age, date of admission and discharge, presenting symptoms, temperature, C-reactive protein (CRP), white blood cell (WBC), CT findings, type and the date of treatment (antibiotics, ultrasound-guided drainage, and surgery), and readmissions. The total numbers of admissions and complications occurring in patients presenting with AUD diagnosed during the study period were registered. The follow-up in terms of recurrence or surgery for all patients in the study population with AUD was at least 1 year.

The study was approved by the Ethics Committee of the Faculty of Medicine, Uppsala University and followed the Declaration of Helsinki guidelines (D.nr2015/213).

### Statistical analysis

Descriptive statistics were used for the analysis of clinical outcomes. Patients with first-time AUD or previous history of diverticulitis were compared. Differences in proportions were calculated using the chi-square test or the *t* test for independent samples. An exact Fisher test was used for small samples. The association between complications and first-time or previous history of diverticulitis was examined using binary multivariate logistic regression model adjusted for age, gender, temperature, WBC, CRP, diabetes mellitus, and immunosuppressive therapy. A *P* value < 0.05 was considered statistically significant. Data were analyzed using the Statistical Package for Social Sciences (SPSS) (version 24; IBM SPSS Inc., Armonk, NY, USA).

### Study population

In total, 1353 episodes of acute diverticulitis were identified in 1188 patients: 218 (18.4%) patients with initially acute complicated diverticulitis, and 970 (81.6%) patients with AUD. The diagnosis was verified by CT scan imaging in 809 (83.4%) of the patients with AUD and constituted the study cohort.

## Results

Of 809 patients with AUD, 642 (79.4%) had first-time AUD and 167 (20.6%) had a previous history of AUD (Table 1). There were no differences in demographic or clinical characteristics such as age, gender, WBC, CRP, or comorbidities between the two groups.

**Table 1:** Demographic data and patient characteristics in CT-verified acute uncomplicated diverticulitis according to index presentation

	First-time diverticulitis *n* = 642	Recurrent diverticulitis *n* = 167	*P*
Age (mean year ± SD)	58.8 ± 13.4	60.3 ± 12.9	0.197
Female (%)	398 (62.0)	108 (64.7)	0.532
Immunosuppressive therapy	25 (3.9)	12 (7.2)	0.094
Diabetes	30 (4.7)	5 (3.0)	0.525
WBC × 10^9^ (mean ± SD)	11.2 ± 5.1	11.6 ± 6.3	0.430
CRP mg/L (mean ± SD)	83.8 ± 56.7	81.0 ± 58.9	0.581
Body temperature (°C) (mean ± SD)	37.1 ± 0.7	37.3 ± 0.7	0.012
Antibiotic therapy (%)	49 (7.6)	16 (9.6)	0.425
Outpatient management (%)	247 (38.5)	40 (24.0)	0.001

In total, 16 (2.0%) patients developed a complication within 1 month with no difference between patients with first-time AUD (11 patients) or previous history of diverticulitis (5 patients; *P* = 0.524). In patients with first-time AUD, 36 (5.6%) had a recurrence within 1 year compared to 28 (16.8%) in patients with a previous history of diverticulitis (*P* < 0.001). Nine (1.4%) patients with first-time AUD had a bowel resection compared to 12 (7.2%) in patients with a previous history of diverticulitis (*P* < 0.001) (Table 2). In the binary logistic regression analysis, first-time diverticulitis was not associated with an increased risk of complications (OR 1.58; CI 0.52–4.81). Age (OR 1.04; CI 1.00–1.08) and immunosuppressive treatment (OR 4.71; CI 1.21–18.28) were associated with increased risk of complications.

**Table 2:** Complications and surgery following CT-verified acute uncomplicated diverticulitis

	First-time diverticulitis *n* = 642	Recurrent diverticulitis *n* = 167	*P*
Complications within 1 month (%)	11 (1.7)	5 (3.0)	0.345
Recurrence within 1 year (%)	36 (5.6)	28 (16.8)	< 0.001
Surgery during follow-up (%)	9 (1.4)	12 (7.2)	< 0.001

Overall, 39% of patients with first-time AUD were managed as outpatients compared to 24% with a previous history of diverticulitis (*P* = 0.001) (Table 1). In the time-trend analysis, the shift toward outpatient management increases from 7% in 2010 to 61% in 2014. The rate of antibiotic therapy was about 7–10% during this time period (Table 3).

**Table 3:** Management of patients with CT-verified acute uncomplicated diverticulitis 2010–2014 (*n* = 809)

	2010 *n* = 142	2011 *n* = 180	2012 *n* = 170	2013 *n* = 173	2014 *n* = 144
Antibiotic therapy (%)	10 (7.0)	15 (8.3)	17 (10.0)	13 (7.5)	10 (6.9)
Outpatient management (%)	10 (7.0)	11 (6.1)	72 (42.4)	106 (61.3)	88 (61.1)

## Discussion

In this large population-based cohort of AUD patients, the risk of complication after an episode of AUD is around 2%, with no difference between patients with first-time AUD and those with a previous history of diverticulitis.

First-time diverticulitis has been considered by some to have a more complicated course [6, 7]. A reason might be the different definitions used for diverticulitis and the different end points for treatment failure in the studies presented. There are two randomized trials on conservative versus antibiotic treatment of AUD with one major difference in patient selection [1, 8]. In the AVOD trial, patients with a previous history of diverticulitis were also included and it was criticized for having included a cohort of patients with less risk of complications. However, the present study shows that few patients with AUD will develop a complication and the risk is not higher for those with first-time AUD.

Patients with a previous history of AUD had an increased risk of recurrence within 1 year compared to those with first-time AUD, but the risk of complications was still low for the patients with recurrent diverticulitis. For the majority of AUD patients, the disease is a self-healing condition without the need for antibiotic therapy or hospitalization, which is consistent with previous reports [3, 5, 9].

In this large population-based cohort of AUD patients, immunosuppressive treatment was found to be associated with an increased risk of complications as has also been reported by others [6, 10]. Hence, patients receiving immunosuppressive treatment should be monitored closely.

The retrospective design of the present study has some inherent limitations. One of the limitations is that only patients with CT-verified AUD were included. In a separate analysis, the results were not altered significantly, when patients with a clinical diagnosis of AUD were included (data not shown). In addition, patients with milder symptoms might have been treated ambulatory by family doctors and data on recurrence for patients moving out of the county were not available. One of the strengths of this study is the population-based design included all patients in the region with acute abdomen.

In conclusion, the result from the current study strengthens the existing evidence of the benign disease course of AUD. Complications develop in a small percentage of the patients with no difference between those with first-time AUD and previous history of diverticulitis.
